# Secreted frizzled-related protein promotors are hypermethylated in cutaneous squamous carcinoma compared with normal epidermis

**DOI:** 10.1186/s12885-015-1650-x

**Published:** 2015-09-22

**Authors:** Junqin Liang, Xiaojing Kang, Yilinuer Halifu, Xuewen Zeng, Tianbo Jin, Mingxia Zhang, Dong Luo, Yuan Ding, Yunmin Zhou, Buwajier Yakeya, Dilinuer Abudu, Xiongming Pu

**Affiliations:** 1 Department of Dermatology, The People’s Hospital of Xinjiang Uyghur Autonomous Region, Urumqi, 830000 China; 2 Department of Plastic surgery, The People’s Hospital of Xinjiang Uyghur Autonomous Region, Urumchi, 830000 China; 3 School of Life Sciences, Northwest University, Xi’an, 710069 China; 4 National Engineering Research Center for Miniaturized Detection Systems, Xi’an, 710069 China

**Keywords:** Cutaneous squamous cell carcinoma, Signaling pathway, DNA methylation, *SFRP*

## Abstract

**Background:**

The Wnt signaling pathway is abnormally activated in many human cancers. Secreted frizzled-related proteins (SFRPs) function as negative regulators of Wnt signaling and play an important role in carcinogenesis. *SFRP* promoter hypermethylation has often been identified in human cancers; however, the precise role of *SFRPs* in cutaneous squamous cell carcinoma (SCC) is unclear.

**Methods:**

The methylation status of the *SFRP* family was analyzed in an age-and sex-matched case-control study, including 40 cutaneous SCC cases and 40 normal controls, using the MassARRAY EpiTYPER system.

**Results:**

The methylation rate of *SFRP1*, *SFRP2*, *SFRP4*, and *SFRP5* promoters was significantly higher in cutaneous SCC tissues than in adjacent tissue and normal skin samples.

**Discussion:**

Our manuscript mainly discussed the average methylation rate of SFRPs (SFRP1, SFRP2, SFRP4, and SFRP5) promoters are significantly high in tumor tissue samples and the average CpG island methylation rate among different pathological levels of cutaneous SCC between these genes are different.

**Conclusions:**

Our findings suggest that promoter hypermethylation of *SFRPs* is associated with the development of carcinoma, and could be a useful tumor marker for cutaneous SCC and other types of cancers.

## Background

Non-melanoma skin carcinomas, comprising cutaneous squamous cell carcinoma (SCC) and basal cell carcinoma (BCC), are the most common malignancies in fair-skinned Caucasians [1]. Unlike almost all basal cell carcinomas, cutaneous SCCs are associated with a substantial risk of metastasis [2]. The etiology of cutaneous SCC is multifactorial, with both environmental and host factors being important. However, exposure to ultraviolet radiation is the most common cause [3, 4]. Other risk factors include impaired immune surveillance, as seen in organ transplant recipients, and human papillomavirus infections [5].

The Wnt family of proteins includes a wide range of secreted growth factors that regulate cell differentiation, proliferation, migration, and organogenesis during embryonic development [6]. Aberrant activation of the Wnt pathway has been observed to inhibit tumor cell apoptosis during human carcinogenesis [7, 8]. Wnt signaling is activated on binding of the Wnt ligand to the frizzled membrane receptor [9], and a family of five secreted frizzled-related glycoproteins (SFRP1-5) has been identified as modulators of Wnt signaling [10]. Moreover, the epigenetic silencing of SFRP genes, leading to oncogenic activation of the Wnt pathway and tumor progression was reported in many human cancers [11–14].

Although the involvement of Wnt signaling in carcinogenesis has been extensively studied, the associations of the *SFRP* family with tumorigenesis have only recently begun to be explored. Previous studies have reported *SFRP* downregulation in colorectal cancer, gastric cancer [15], and invasive breast tumors [16, 17]. Additionally, Wnt5a signaling was found to contribute to tissue invasion by non-melanoma skin cancer, including both SCC and BCC [18]. Although non-melanoma skin cancer is common, the incidence in China is very low, and differences have been demonstrated between Caucasians and Hong Kong Chinese patients regarding disease features [19]. In the present study, we used the Mass ARRAY EpiTYPER system to examine the methylation status of *SFRP* family members, including *SFRP1*, *SFRP2*, *SFRP4*, and *SFRP5* in different tissue samples from a Chinese population of the Xinjiang Uygur Autonomous Region.

## Methods

### Study populations and tissue samples

To investigate the methylation status of the *SFRP* family, we conducted an age- and gender- matched case-control study including 40 cutaneous SCC cases and 40 normal controls. The patients were recruited from the Dermatology Department of the People’s Hospital of Xinjiang Uygur Autonomous Region (Xinjiang, China), and had received a diagnosis of histologically confirmed cutaneous SCC between January 2012 and February 2014. Controls were normal individuals from the Plastic Surgery Department of the hospital who reported no history of any type of cancer at the time of study recruitment. The basic characteristics for the participants included in this study are presented in Table 1. Cutaneous SCC tissues (*n* = 40) and adjacent tissues (*n* = 40) were obtained from the head or the hands/legs of the 40 cutaneous SCC patients. Normal skin samples (*n* = 40) were collected from the face or the hands/legs of the 40 controls. This study was approved by the ethics committee of the People’s Hospital of Xinjiang Uygur Autonomous Region. Written informed consent was also obtained from each participant enrolled in the study.

**Table 1:** Basic characteristics of the cases and controls in this study

Variables	Case (*N* = 40)	Control (*N* = 40)	Total	*p*-value
Sex, No. (%)				0.749*
Male	21 (52.50)	22 (55.00)	43	
Female	19 (47.50)	18 (45.00)	37	
	40	40		
Grade, No. (%)				
Stage I	21 (52.50)			
Stage II	12 (30.00)			
Stage III	7 (17.50)			
Stage IV	0			
Mean age ± SD	67.11 ± 9.24	64.31 ± 8.91		0.707**

*P values were calculated from two-sided chi-squared tests

**P values were calculated by Student’s *t*-tests

### DNA extraction, sodium bisulfite modification, and PCR

Genomic DNA from tissue samples was extracted using the Tissue DNA Kit (Qiagen) according to the manufacturer’s recommendations. The DNA concentration and quality were measured using the NanoDrop ND-1000 spectrophotometer (NanoDrop Technologies, Houston, TX). Bisulfite modification of genomic DNA was performed using the EZ DNA Methylation Kit (Zymo Research) according to the manufacturer’s protocol. Bisulfite-treated genomic DNA was amplified in a 384-well plate using HotStar Taq Polymerase in a 5-μl reaction volume (Qiagen). PCR conditions were 94 °C for 4 min followed by 45 cycles of 95 °C for 20 s, 56 °C for 20 s, and 72 °C for 60 s, with a final extension of 72 °C for 3 min. Primer sequences are given in Table 2.

**Table 2:** Primer sequences for amplifying *SFRP* genes

Gene		Forward	Reverse	bp	CpGs	Tm
SFRP1	SFRP1_1	aggaagagagTATGTGTGTTTGAGTGATGGATTTG	cagtaatacgactcactatagggagaaggctAAACAACACCTCTCCAAATAAAACC	290	7	56
	SFRP1_2	aggaagagagAGTTTGGGAGGTTAAGGTAGGAGTA	cagtaatacgactcactatagggagaaggctAAAACCTAAATCATACTTACAAACCCAT	300	12	56
SFRP2	SFRP2_1	aggaagagagGGTTAGGTTTTTTTGTTTGTTGTTT	cagtaatacgactcactatagggagaaggctCACAACCAAAATTTTCTTAACCTTT	235	9	56
	SFRP2_2	aggaagagagGGGGATGAATGAGTTAATTTTAGTT	cagtaatacgactcactatagggagaaggctAAAAATCTCACCAATCACACAAAAC	296	9	56
SFRP4	SFRP4_1	aggaagagagGTATGTGTGTTTGAGTGATGGATTT	cagtaatacgactcactatagggagaaggctTCCCAACTAACAAAAATTCAAAAAA	314	6	56
	SFRP4_2	aggaagagagTTTTGAATTTTTGTTAGTTGGGAAA	cagtaatacgactcactatagggagaaggctAAAACCTAAATCATACTTACAAACCCA	390	36	56
SFRP5	SFRP5_1	aggaagagagGTATGTGTGTTTGAGTGATGGATTT	cagtaatacgactcactatagggagaaggctTCCCAACTAACAAAAATTCAAAAAA	323	6	56
	SFRP5_2	aggaagagagTTTTGAATTTTTGTTAGTTGGGAAA	cagtaatacgactcactatagggagaaggctAAAACCTAAATCATACTTACAAACCCA	329	6	56

SFRP_1 and SFRP_2 refer to two amplified fragments of *SFRP*

### Quantitative DNA methylation analysis by MassARRAY EpiTyper

The PCR products were treated according to the standard protocol (Sequenom EpiTyper Assay) by SAP treatment and T-cleavage reaction. The samples were then cleaned by Resin and were dispensed to a 384 SpectroCHIP by Nanodispenser. DNA methylation levels were determined as previously described [20] using MassARRAY EpiTYPER (SEQUENOM Inc., Herston, QLD, Australia) system, which is based on MALDI-TOF MS [21]. The mass spectrum was collected by MassARRAY Spectrometer and analyzed by EpiTYPER v.1.0 software (SEQUENOM).

### Statistical analysis

The SPSS 17.0 statistical software (SPSS Inc., Chicago, IL, USA) and Microsoft Excel (Microsoft Corporation, Redmond, WA, USA) were used for statistical analysis. The methylation levels in cutaneous SCC patients and normal samples were compared by nonparametric Mann-Whitney *U* test or the Kruskal-Wallis *H* test. All *P*-values presented in this study were two sided, and we used *P < 0.05* as the cutoff value for statistical significance. The receiver operating characteristic (ROC) curve was used to estimate the specificity, sensitivity, and accuracy of the regression test.

## Results

In the present study, we investigated the methylation status of *SFRP1*, *SFRP2*, *SFRP4*, and *SFRP5* in 40 cutaneous SCC patients and 40 normal subjects. Table 1 showed the distribution of age, sex, and pathological level among cases and controls. The mean age for the case group was 67.11 ± 9.24 years and 64.31 ± 8.91 years for the control group. There were no significant differences in age and sex distribution between the case and control groups (*p* > 0.05).

We investigated the promoter methylation rate of *SFRP1*, *SFRP2*, *SFRP4*, and *SFRP5* in 40 cutaneous SCC tissues, 40 adjacent tissues, and 40 normal samples using the MassARRAY EpiTYPER system. Aberrant promoter methylation of *SFRP1*, *SFRP2*, *SFRP4*, and *SFRP5* was detected in cutaneous SCC tissues and adjacent tissues compared with normal samples. Additionally, the frequency of *SFRP* promoter methylation in tumors was significantly higher than in adjacent tissues and normal samples (Table 3).

**Table 3:** Methylation frequency (%) comparison of *SFRP* CpG sites in three tissue groups

Genes	No. of CpG islands	Frequency of promoter methylation (%)		
		T vs. N	T vs. A	A vs. N
SFRP1	17	82.35 (14/17)	58.82 (10/17)	29.41 (5/17)
SFRP2	14	50 (7/14)	57.14 (8/14)	42.86 (6/14)
SFRP4	18	72.22 (13/18)	72.22 (13/18)	61.11 (11/18)
SFRP5	11	54.54 (6/11)	54.54 (6/11)	54.54 (6/11)
P*-value		<0.05	<0.05	<0.05

*T* tumor tissues; *A* adjacent tissues; *N* normal samples

*Nonparametric Mann-Whitney *U* test

**Fig. 1:**
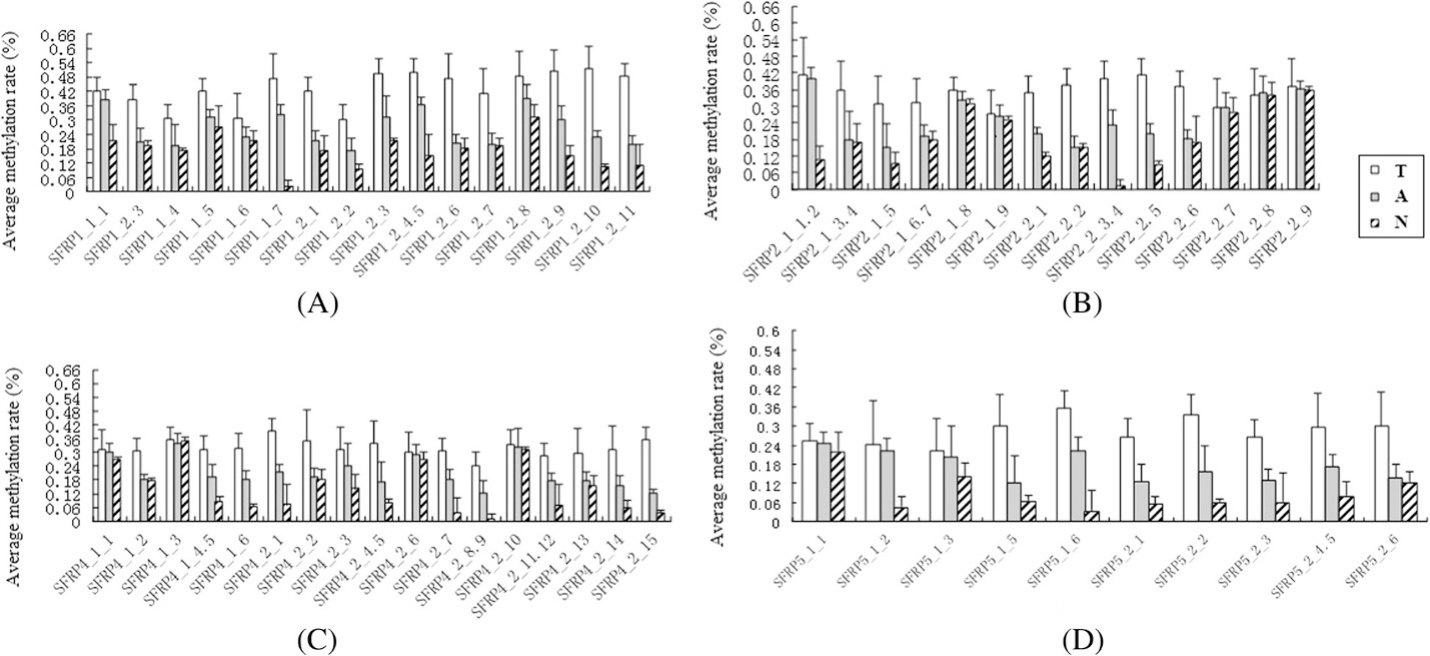
Comparison of average CpG island methylation rate in the *SFRP* gene family. T, tumor tissues; A, adjacent to carcinoma tissue; N, normal tissue samples

We also assessed the average CpG island methylation rate of *SFRPs* among different pathological levels of cutaneous SCC. We observed a significant increase in the methylation rate of different *SFRP* CpG islands with higher pathological levels (Table 4). CpG island methylation of different *SFRPs* was shown to vary among different tissues. The methylation rate was also much higher in cutaneous SCC tissues than in adjacent tissues, but was lower in normal control samples (Fig. 1a–d).

**Table 4:** Methylation frequency (%) of *SFRP* CpG islands at different cutaneous SCC pathological levels

Gene	CpG Site	Stage I		Stage II		Stage III		H	*P*-value
		N	$\overline{x}\pm s$	N	$\overline{x}\pm s$	N	$\overline{x}\pm s$		
SFRP1	CpG1_5	0	0.4012 ± 0.098	0	0.4521 ± 0.115	9	0.4952 ± 0.109	6.157	0.024*
	CpG1_7	9	0.4011 ± 0.134	6	0.4811 ± 0.024	2	0.5121 ± 0.124	5.103	0.031
	CpG2_1	7	0.4141 ± 0.19	0	0.4267 ± 0.314	2	0.4013 ± 0.217	2.136	0.021
	CpG2_8	5	0.4231 ± 0.824	7	0.4501 ± 0.224	4	0.5011 ± 0.523	8.514	0.017
SFRP2	CpG1_5	44	0.3329 ± 0.101	29	0.3796 ± 0.170	19	0.4241 ± 0.183	6.142	0.015
	CpG2_1	47	0.4094 ± 0.036	48	0.4367 ± 0.316	17	0.4713 ± 0.107	6.136	0.023
	CpG2_3.4	55	0.4017 ± 0.098	42	0.4406 ± 0.194	18	0.4891 ± 0.153	5.218	0.015
SFRP4	CpG1_3	2	0.3029 ± 0.089	3	0.3496 ± 0.012	2	0.3867 ± 0.083	7.158	0.009
	CpG2_2	9	0.2994 ± 0.037	2	0.3367 ± 0.016	5	0.4113 ± 0.086	7.156	0.023
SFRP5	CpG1_5	40	0.3014 ± 0.052	33	0.3834 ± 0.021	18	0.4345 ± 0.037	5.54	0.031
	CpG2_1	32	0.2941 ± 0.098	19	0.3167 ± 0.314	34	0.4013 ± 0.057	3.136	0.021

N: number of samples; H: the Kruskal-Wallis *H* test

*p ≤ 0.05 indicates statistical significance

**Fig. 2:**
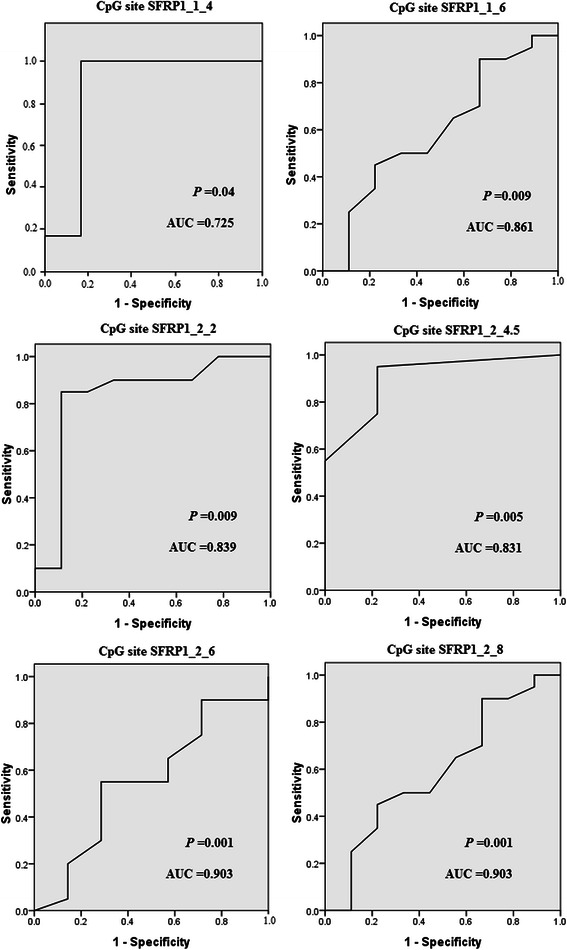
Receiver operating characteristic (ROC) curves for six CpG sites in the *SFRP1* promoter. The ROC curves plot sensitivity and 1-specificity. Areas under the curve (AUC) and *P* values were shown in the graph

Finally, we performed ROC curve analysis of *SFRP1* CpG island methylation (Fig. 2). ROC curve is a graphic presentation of the relationship between both sensitivity and specificity, and it helps to decide the optimal model through determining the best threshold for a diagnostic/predictive test [22]. The area under the curve (AUC) of the ROC curves provides a way to measure the accuracy of a diagnostic/predictive test. The larger the area, the more accurate the test is [22]. As shown in Fig. 2, all these six CpG sites showed high AUC of the ROC curves, ranging from 0.725 to 0.903. These results indicate that the detection of *SFRP1* CpG methylation may be used as an early biomarker for SCC diagnosis.

## Discussion

Cutaneous SCC is the second most common cancer among Caucasians [2]. Although it has been suggested to arise through the accumulation of multiple genetic changes involving a complex multi-step process [1], the precise pathogenesis remains unclear. However, numerous studies have recently shown that the epigenetic dysregulation of tumor suppressor genes could serve as a biomarker to predict the diagnosis and prognosis of human disease and malignancy [23–25].

In the present study, we describe the aberrant DNA methylation of four members of the *SFRP* family, *SFRP1*, *SFRP2*, *SFRP4*, and *SFRP5*, in cutaneous SCC in a Chinese population. Methylation of these genes has been reported previously in lung cancer, prostate cancer, colorectal cancer [26], and human glioblastoma [27]. Additionally, several studies have reported the lack of methylation in nonmalignant tissues and described methylation as a tumor-restricted event [23, 28, 29]. Here, we showed that frequent promoter hypermethylation of *SFRPs* was significantly elevated in cutaneous SCC samples compared with adjacent tissues and control samples. Interestingly, we also observed marked differences in the levels of aberrant DNA methylation between these genes (*SFRP1, SFRP2, SFRP4,* and *SFRP5*), suggesting that hypermethylation of these *SFRPs*, particularly of *SFRP1*, could be important in the onset of cutaneous SCC.

Aberrant hypermethylation of CpG islands in gene promoters was previously shown to be a primary mechanism in the inactivation of several tumor suppressor genes [30]. We assessed the methylation status of the CpG islands in *SFRP* promoters in different pathological levels of cutaneous SCC tissue, and found the methylation rate to increase with the pathological level. ROC curve analysis showed that the *SFRP1* CpG site is a potential biomarker of cutaneous SCC, and the further identification of genes that are methylated during cutaneous SCC carcinogenesis together with an improvement in the quantitative rather than the qualitative analysis of methylation will be of great value in the future molecular screening of cutaneous SCC.

The potential importance of *SFRP* silencing in cutaneous SCC relates to its involvement in the Wnt signaling pathway. This pathway is involved in the tumorigenesis of many human cancers, including those of the colon, breast, lung, and liver, as well as melanomas [7]. Chung et al. also recently demonstrated the frequent aberrant methylation of *SFRPs* in cervical SCC [31]. Additionally, two further studies indicated that frequent aberrant methylation of *SFRPs* occurs in hepatocellular carcinoma, and that restoration of *SFRP1* attenuates Wnt signaling, decreases the abnormal accumulation of β-catenin in the nucleus, and suppresses cell growth [32, 33]. To date, however, the regulation of the Wnt pathway in cutaneous SCC has remained unclear. Pourreyron et al. [18] demonstrated that Wnt5a is overexpressed in non-melanoma skin cancer, which contributes to tissue invasion. Additionally, the study presented here also suggests the upregulation of *SFRP1* and *SFRP*2 expression in cutaneous SCC, in particular *SFRP2*, exhibit very high constitutive expression in normal skin. The data provided by the Human Protein Atlas on cutaneous SCC (www.proteinatlas.org) showing that the *SFRPs* (*SFRP1*, *SFRP2*, *SFRP4*, and *SFRP5*) exhibit very low expression in cancer tissues, except *SFRP1*, which was slightly high. Our results showed that the *SFRPs* were highly methylated in cutaneous SCC, which in accordance with the data of the Human Protein Atlas on cutaneous SCC. Nevertheless, much work remains to be done to obtain a clearer understanding of the role of *SFRPs* in Wnt signaling and tumor pathogenesis.

## Conclusions

In conclusion, our study demonstrates that promoter hypermethylation of *SFRPs* (*SFRP1*, *SFRP2*, *SFRP4*, and *SFRP5*) is associated with the development of cutaneous SCC. Although it is not possible to determine whether *SFRPs* are directly related to carcinogenesis from this study, the aberrant methylation of *SFRPs* may nevertheless be a useful tumor biomarker for early diagnosis. Despite the current study possessing enough statistical power, some limitations should be considered. First, we limited our investigation to only four *SFRPs*: *SFRP1*, *SFRP2*, *SFRP4*, and *SFRP5*. Therefore, we cannot exclude the possibility that other genes may play a role in the pathogenesis of cutaneous SCC. Second, the sample size of our study was relatively small, so our findings should be confirmed in larger case-control studies.

## Abbreviations

SCC: Squamous cell carcinoma
BCC: Basal cell carcinoma
SFRPs: Secreted frizzled-related proteins
ROC: Receiver operating characteristic
AUC: Area under the curve
